# Vascular Adhesion Protein 1 in the Eye

**DOI:** 10.1155/2013/925267

**Published:** 2013-06-04

**Authors:** Wenting Luo, Fang Xie, Zhongyu Zhang, Dawei Sun

**Affiliations:** ^1^Department of Ophthalmology, 2nd Affiliated Hospital of Harbin Medical University, 246 Xuefu Road, Harbin 150001, China; ^2^Harbin Medical University-The Key Laboratory of Myocardial Ischemia, Chinese Ministry of Education, Harbin 150001, China; ^3^Department of Ophthalmology, 1st Affiliated Hospital of Harbin Medical University, Harbin 150001, China

## Abstract

Semicarbazide-sensitive amine oxidase/vascular adhesion protein-1 (SSAO/VAP-1), a dual-function molecule with adhesive and enzymatic properties, is expressed on the surface of vascular endothelial cells of mammals. It also exists as a soluble form (sVAP-1), which is implicated in oxidative stress via its enzymatic activity and can be a prognostic biomarker. Recent evidence suggests that VAP-1 is an important therapeutic target for several inflammation-related ocular diseases, such as uveitis, age-related macular degeneration (AMD), and diabetic retinopathy (DR), by involving in the recruitment of leukocytes at sites of inflammation. Furthermore, VAP-1 plays an important role in the pathogenesis of conjunctival inflammatory diseases such as pyogenic granulomas and the progression of conjunctival lymphoma. VAP-1 may be an alternative therapeutic target in ocular diseases. The in vivo imaging of inflammation using VAP-1 as a target molecule is a novel approach with a potential for early detection and characterization of inflammatory diseases. This paper reviews the critical roles of VAP-1 in ophthalmological diseases which may provide a novel research direction or a potent therapeutic strategy.

## 1. Introduction

Vascular adhesion protein-1 (VAP-1) is a homodimeric sialylated glycoprotein originally discovered in inflamed synovial vessels by Salmi and Jalkanen in 1992 [1]. VAP-1 is a multifunctional molecule that possesses enzymatic activity known as semicarbazide-sensitive amine oxidase (SSAO) and is involved in the leukocyte recruitment cascade. The VAP-1 molecule consists of an extracellular part, which harbors the catalytic site, a transmembrane segment, and a short intracellular N-terminal tail [2, 3]. On the plasma membrane, VAP-1 normally forms a homodimer of two 90 kDa glycoproteins. The extracellular part of each monomer consists of three domains (D2–D4). VAP-1 has a relatively narrow substrate channel formed by domains D4 and D3, and a key leucine (469 in human) guards the entry of substrates. The large D4 domains, from each subunit, form the dimer interface and each also contains a catalytic site, buried at the base of a deep cleft.

VAP-1 exists as membrane-bound and soluble forms in the plasma. Its major sources are endothelial cells, smooth muscle cells, and the adipocytes [4]. VAP-1 is expressed on the endothelium of human tissues such as skin, brain, lung, liver, and heart under both normal and inflamed conditions [4–8]. In the ocular tissues of humans and rats, VAP-1 is localized on the endothelial cells of retinal and choroidal vessels [9–12]. VAP-1 labeling showed the highest intensity in both arteries and veins of neuronal tissues: retina and optic nerve, the moderate intensity in scleral and choroidal vessels, and the lowest intensity in the iris vasculature [10]. Moreover, VAP-1 intensity was significantly higher in the arteries compared to veins [10]. 

**Figure 1:**
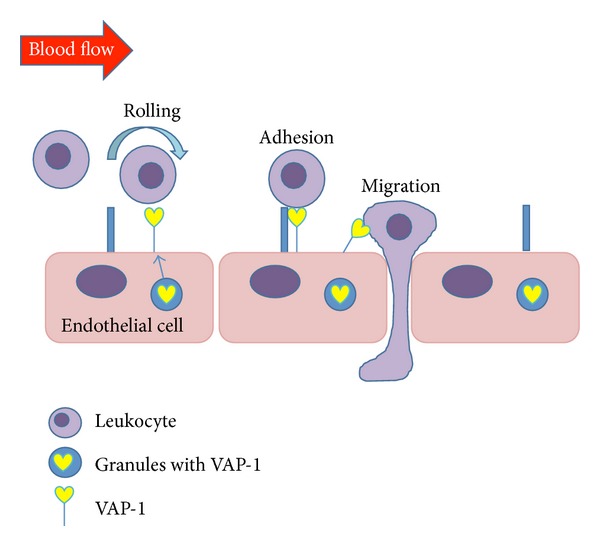
Under normal conditions, VAP-1 is mainly absent from the endothelial cell surface and is stored within intracellular granules, while on inflammation, it is rapidly translocated to the endothelial cell surface and facilitates the recruitment of leukocytes into the inflamed tissues together with other leukocyte adhesion molecules.

Under normal conditions, VAP-1 is mainly absent from the endothelial cell surface and is stored within intracellular granules, while on inflammation, it is rapidly translocated to the endothelial cell surface and facilitates the recruitment of leukocytes into the inflamed tissues together with other leukocyte adhesion molecules [13] (Figure 1). In fact, previous studies have elucidated that VAP-1 is involved in the molecular mechanisms of acute ocular inflammation [11], inflammation-associated ocular angiogenesis [12], and leukostasis under diabetic conditions [10]. Indeed, VAP-1 inhibition may be a novel and potent therapeutic strategy in the treatment of ocular inflammatory diseases. Notably, SSAO/VAP-1 contributes to inflammation not only through its role as an adhesion molecule but also through its function as an enzyme by causing the formation of cytotoxic molecules such as hydrogen peroxide, aldehyde, and ammonia [14]. These molecules are involved in the pathophysiology of ocular inflammation [15, 16], and their inhibition, for instance, through antioxidants, recovers the integrity of the blood-aqueous barrier in endotoxin-induced uveitis (EIU) animals [17]. 

Here we give an overview on the new research progresses of VAP-1 in the ocular diseases including uveitis, age-related macular degeneration (AMD), diabetic retinopathy (DR), and ocular tumor. The connection between VAP-1 and ocular diseases will be elucidated and may provide a new research direction for the diagnosis and treatment of these ocular diseases.

## 2. VAP-1 in Acute Inflammation of Endotoxin-Induced Uveitis 

Uveitis is regarded as a sight-threatening disease. Complications such as cystoid macular edema, glaucoma, vascular occlusion, and proliferative vitreoretinopathy are common causes of permanent vision loss [18–21]. EIU is one of animal models to establish new therapeutic targets for treating human uveitis, which is marked by a vasodilatation of the iris and vascular changes in the ciliary body, accompanied by an increased vascular permeability and breakdown of the blood-aqueous barrier [22–24]. The leukocytes infiltrate into the anterior chamber, vitreous cavity, and retina from ciliary body and iris in conjunction with protein extravasation into the aqueous humor. As part of this inflammatory response, endothelial adhesion molecules are upregulated. For example, endothelial P-selectin, which mediates the first step of the leukocyte recruitment, the tethering, and rolling, is upregulated in retinal vessels of EIU animals [25, 26]. Furthermore, intercellular adhesion molecule-1 (ICAM-1), which mediates the subsequent step of firm leukocyte adhesion to the vascular endothelium, is increased in the retina of EIU animals [27, 28]. Functional inhibition of P-selectin [29] or ICAM-1 [28] prevents the infiltration of leukocytes into the inflamed ocular tissues during EIU, and thus attenuates the inflammatory response at the early stages of rolling and firm adhesion. 

In 2008, Noda et al. investigated the role of VAP-1 in an established model of EIU. VAP-1 is constitutively expressed in the normal retina, and its expression is elevated together with SSAO activity during EIU [11]. Their data also indicate that VAP-1 inhibition substantially suppresses retinal inflammation during EIU on a molecular, cellular, and organ level. For example, VAP-1 inhibition in EIU animals significantly suppressed leukocytes recruitment to the anterior chamber, vitreous, and retina, as well as retinal endothelial P-selectin expression. The diameter of the retinal veins and arteries of EIU animals, 24 h after LPS injection, was significantly larger than the corresponding retinal vessels in normal animals. However, VAP-1 inhibition reduced the diameter of corresponding retinal veins and arteries 24 h after LPS injection, compared with vehicle-treated rats even though the difference did not reach statistical significance. To sum up, VAP-1 is crucially involved in leukocyte infiltration into ocular tissues during acute inflammation of EIU. VAP-1 inhibition may even prevent leukocyte recruitment at the early stage of rolling and become a novel strategy in the treatment of uveitis (Table 1).

## 3. VAP-1 in the Choroidal Neovascularization 

Choroidal neovascularization (CNV) is the main cause of severe vision loss in patients with age-related macular degeneration (AMD) [30]. Inflammation plays a critical role in the formation of CNV lesions and may contribute to the pathogenesis of both the nonexudative and exudative forms of AMD [31, 32]. For example, inflammatory cells are found in surgically excised CNV lesions from AMD patients [33–36] and in autopsied eyes with CNV [37–39]. In particular, macrophages have been implicated in the pathogenesis of AMD due to their spatiotemporal distribution in the proximity of the CNV lesions in experimental models and humans [40–42]. Macrophages are a source of proangiogenic and inflammatory cytokines, such as vascular endothelial growth factor (VEGF) [43] and tumor necrosis factor (TNF)-*α* [44], both of which significantly contribute to the pathogenesis of CNV [45, 46]. Furthermore, druse which has proven to be one of the earliest signs of AMD contains many inflammatory molecules [47, 48]. Some inflammatory molecules such as the complement components C3a and C5a are proinflammatory and can induce VEGF [49].

As an endothelial adhesion molecule involved in leukocyte recruitment under inflammatory conditions, VAP-1 was recently showed to contribute to the recruitment of macrophages to CNV lesions in a rat laser-induced AMD model and had a novel link with angiogenesis [12]. In their study, VAP-1 was found to be expressed in the choroid and retina, exclusively in the vessels, and localized in the vessels of the CNV lesions. Inhibition of VAP-1 significantly decreased CNV size, fluorescein angiography leakage, and the accumulation of macrophages in CNV lesions [12]. Furthermore, VAP-1 blockade significantly reduced the expression of inflammation-associated molecules such as tumor necrosis factor (TNF)-*α*, monocyte chemoattractant protein (MCP)-1, and intercellular adhesion molecule (ICAM)-1 [12].

Most recently, in a mouse laser-induced CNV model, VAP-1 inhibition significantly attenuated CNV formation in a dose-dependent manner and reduced macrophage infiltration into CNV lesions [50]. Furthermore, VAP-1 blockade decreased the expression of ICAM-1 and MCP-1, both of which played a pivotal role in macrophage recruitment [50]. Thus, VAP-1 blockade reduced macrophage recruitment into CNV lesion indirectly via suppression of other adhesion molecules. Previous studies have demonstrated that marked suppression of VEGF is crucial for the suppression of CNV formation in the laser-induced CNV model [51, 52]. However, in this study VAP-1 blockade showed weak inhibitory effects on VEGF, a key molecule for angiogenesis, whereas CNV formation was significantly suppressed. It may indicate that VAP-1 inhibition ameliorates ocular angiogenesis through mechanism(s) other than VEGF expression. Further evaluation is needed to elucidate the detailed mechanism(s). In conclusion, the current data suggest that VAP-1 may be an attractive molecular target in the treatment of CNV formation of AMD (Table 1). 

## 4. VAP-1 in Chronic Low-Grade Inflammation of Diabetic Retinopathy

Diabetic retinopathy (DR) is one of the main microvascular complications of diabetes and a leading cause of adult vision loss [53, 54]. Recent studies have elucidated that chronic, low-grade inflammation underlies much of the vascular complications of DR [55, 56]. Many molecular and functional changes that are characteristics of inflammation have been detected in DR. The recruitment of leukocytes has been found to be significantly increased in retinas of diabetic animals [57–59] and might contribute to the capillary nonperfusion of diabetic retinopathy. Leukocytes firmly adhering to capillary endothelial cells via adhesion molecules induce apoptotic changes in retinal endothelial cells. 

As demonstrated through several lines of evidence, VAP-1 seems to be a key player in the inflammation associated with DR. In 2009, Noda et al. investigated the role of VAP-1 in DR. Contrastively, retinal VAP-1 expression was higher in diabetic animals compared to the normal controls; however, the difference did not reach statistical significance [10]. Their results also suggested that VAP-1 principally regulated the step of leukocyte transmigration, with little influence on the preceding step of firm adhesion [10]. This provides a clear distinction between the role of VAP-1 in acute and chronic inflammation. During acute inflammation VAP-1 regulates both firm adhesion and transmigration [11], while in chronic low-grade inflammation, such as found during diabetes, VAP-1 may only regulate transmigration. In conclusion, VAP-1 contributes to the inflammatory outcome of DR. VAP-1 inhibition may be beneficial in the treatment and prevention of DR. Further investigation may provide a better understanding of the role of VAP-1 in DR.

VAP-1 also exists as a soluble form in serum which retains its enzymatic function [60]. Like other soluble adhesion molecules, sVAP-1 modulates lymphocyte adherence. In fact, sVAP-1 appears to augment lymphocyte binding to endothelial cells [61]. Much attention has recently been paid to the elevated serum concentration of sVAP-1 in patients with type 1 and type 2 diabetes [61, 62]. In type 2 diabetes, sVAP-1 even serves as an independent prognostic marker for the diabetic complications and predicts the risk for cardiovascular and cancer mortality in these patients [63]. Moreover, patients with DR display significantly higher plasma SSAO activities compared to patients without DR [61] (Table 1).

In a recent clinical study, Murata et al. [64] demonstrated that sVAP-1 is increased and correlated with oxidative stress in the vitreous fluid of patients with PDR. Furthermore, retinal capillary endothelial cells produce the membrane-bound form of VAP-1 and release sVAP-1 when stimulated with high glucose or inflammatory cytokines such as TNF-*α* and IL-1β. MMP-2 (matrix metalloproteinases-2) and MMP-9 can degrade type IV collagen, laminin, and fibronectin, the main constituents of the basement membrane; thereby, MMPs play a crucial role in the degradation of basement membrane during angiogenesis [65, 66]. MMP-2 and MMP-9 are the proteinases predominantly responsible for VAP-1 shedding from retinal capillary endothelial cells [64]. The present data provide evidence on the link between sVAP-1 and type IV collagenases in the pathogenesis of PDR. Therefore, further studies are needed to clarify the relationship between sVAP-1 and other ocular diseases.

## 5. VAP-1 in Ocular Tumor

The relationships between VAP-1 and tumors have been reported. In human skin melanoma, VAP-1 protein expression was significantly decreased in intratumoral vessels [67]. It has been demonstrated that the 5-year survival of melanoma patients with low VAP-1 protein expression in intratumoral blood vessels was lower than that of those patients with high VAP-1 expression [67]. Strong expression of VAP-1 on tumor endothelium could distinguish human hepatocellular carcinoma from colorectal hepatic metastases [68]. Furthermore, some studies indicate that patients with low sVAP-1 levels have significantly worse prognosis of colorectal cancer and that sVAP-1 is an independent marker of hepatic and lymph node metastasis in these patients [69]. A similar correlation with low sVAP-1 and poor prognosis was reported in gastric cancer [70]. 

Lately, Fukuhara et al. examined the immunolocalization of VAP-1 in pyogenic granuloma and extranodal marginal zone B-cell lymphoma (EMZL) as common human conjunctival tumors. They showed strong expression of VAP-1 protein in intratumoral blood vessels of pyogenic granuloma, a benign inflammatory conjunctival tumor, and relatively lower expression in EMZL, a malignant inflammatory tumor [71]. Moreover, the microvessel density was high in pyogenic granuloma compared to that in EMZL [71]. Their data suggest that VAP-1 plays an important role in the pathogenesis and development of conjunctival inflammatory diseases such as pyogenic granulomas, whereas the relatively lower expression of VAP-1 in intratumoral microvessels might be correlated with the progression of conjunctival lymphoma.

Furthermore, VAP-1 is involved in angiogenesis and tumor growth via controlling the migration of Gr-1+CD11b+ myeloid cells, which comprise immature macrophages and dendritic cells playing a pivotal role in tumor angiogenesis [72]. VAP-1 may support tumor progression. VAP-1 deficient mice melanoma and lymphoma tumors grew more slowly than in wild-type animals [72]. The tumors in VAP-1−/− host had defective angiogenesis and impaired recruitment of myeloid-derived suppressor cells (MDSCs). Notably, if the MDSCs were ablated from the mice, VAP-1 deficiency no longer protected the animals. Moreover, genetic experiments with transgenic mice expressing an enzymatically inactive mutant of VAP-1 showed that the effects on MDSC accumulation were dependent on the oxidase activity of VAP-1. Therefore, VAP-1 enhances local malignant lymphoma growth by increasing the recruitment of myeloid leukocytes into the tumors. These data suggest that VAP-1 contributes to the development of conjunctival EMZL. Since tumor cells utilize the catalytic activity of VAP-1 to recruit myeloid cells into tumors, and to support tumor progression, small-molecule VAP-1 inhibitors could be an effective immunotherapy for the inhibition of tumor progression [73]. Currently Salmi and Jalkanen [74] hypothesize that the VAP-1 expressing in neoangiogenic vessels of the tumor bind MDSC. As a consequence, the intratumoral numbers of this particular protumorigenic leukocyte subtype are selectively increased, with a concomitant stimulation of the neoangiogenesis and enhancement of the immunosuppressing gene signature of the tumor microenvironment. In conclusions, VAP-1 may be an alternative therapeutic target in ocular tumors (Table 1).

## 6. The Role of VAP-1 in Molecular Imaging

The special structure of the eye provides a unique opportunity for noninvasive light-based imaging of fundus vasculature. Using adhesion-molecule-conjugated fluorescent microspheres (MSs) in live animals, researchers showed early endothelial changes in ocular microvessels at an early stage [75], which were previously detectable only by the most sensitive in vitro techniques, such as immunohistochemistry or PCR. This novel method also allows evaluation of leukocyte-endothelial interaction in the retinal and choroidal capillaries flow or identification of specific molecular changes during disease. Molecular imaging is defined as the ability to visualize and quantitatively measure the function of biological and cellular processes in vivo [76, 77]. In vivo molecular imaging has a great potential to impact medicine by detecting diseases or screening diseases in early stages, identifying extent of disease, selecting disease- and patient-specific therapeutic treatment, applying a directed or targeted therapy, and measuring molecular-specific effects of treatment. Inflammation and tracing of inflammatory cells have been a key topic in molecular imaging in recent years. An ideal target for in vivo imaging of inflammation would be a molecule that is normally absent from the endothelium of healthy tissues but is induced at the onset of inflammation. 

According to our previous summarization, VAP-1 may be suitable as an imaging target in the diagnosis and treatment of ocular inflammatory diseases. A recent paper using the technique of in vivo molecular imaging showed that VAP-1 was expressed in the resting and angiogenic corneal blood vessel endothelial cells but not in lymphatic vessels [78]. Moreover, the study demonstrated a higher VAP-1 expression in angiogenic than normal blood vessels, which revealed the key role of VAP-1 in angiogenesis-related diseases [78]. In the study, IL-1–induced M2 macrophage infiltration as well as lymph-and angiogenesis were blocked by VAP-1 inhibition, whereas VEGF-A-induced lymph- and angiogenesis were unaffected by VAP-1 inhibition [78]. These results indicate a critical role for VAP-1 in lymph- and angiogenesis-related macrophage recruitment. To sum up, VAP-1 might become a new target for the treatment of inflammatory lymph- and angiogenic diseases, including cancer.

The proof of concept regarding the use of VAP-1 as an imaging target was also obtained with iodinated monoclonal antibodies against VAP-1. They were used to detect skin and joint inflammation in the pig [79]. Currently, VAP-1 was investigated as a potential target for in vivo imaging of inflammation by means of PET [80]. Panning of phage display libraries with recombinant VAP-1 has led to the identification of the first cellular counter-receptors of VAP-1. These experiments showed that VAP-1 binds to Siglec-9 and Siglec-10 proteins both in cell free protein-protein interaction assays and in different cell-based models [80–83]. Siglecs belong to a family of lectin molecules, which bind to sialic acids and mediate various adhesive and signaling events both within the immune system and elsewhere in the body [84]. The cellular distributions of Siglec-9 and -10 are very different: Siglec-9 is expressed on all granulocytes, whereas Siglec-10 is present mainly on B-cells. Based on molecular modeling, it is plausible that both Siglecs can present specific arginine residues into the enzymatic cavity of VAP-1. Although the side chain of arginine terminates in a complex guanidinium structure rather than in normal primary amine, the arginine 293 of Siglec-10 has been experimentally demonstrated to function as a substrate of VAP-1 [81]. Thus, these molecules can apparently serve as surface-bound substrates of VAP-1. Siglec-VAP-1 interaction can be utilized for the imaging of inflammation and cancer in vivo [82]. Short synthetic Siglec-9 peptides (containing the VAP-1 interacting core sequence) localize selectively to sites of inflammation in vivo in VAP-1 expressing transgenic mice but not in VAP-1 deficient mice. From the clinical point of view, a VAP-1-specific imaging agent could be valuable for the detection of infection/inflammation during its early stages. As a diagnostic tool, the method could differentiate between inflammation and cancerous growth or bacterial infection from sterile inflammation [85].

## 7. Conclusions and Future Perspectives

Aberrant leukocyte trafficking to sites of inflammation is often harmful leading to tissue damage. Therefore, molecules responsible for the harmful traffic are theoretically excellent targets to prevent inflammations. VAP-1 acts via direct interactions with its counter-receptors, and more importantly, exerts its effects via the end-products of its enzymatic activity. The inhibitors of VAP-1 may be anti-inflammatory and antiangiogenic agents to decrease the inflammation in ophthalmological diseases. The end-products of VAP-1 are proinflammatory, so they would be beneficial to suppress VAP-1 and alleviate inflammatory reactions. In comparison to other trafficking-associated molecules, VAP-1 provides pharmaceutical industry with unique targets for the design of novel molecule-targeted therapies of inflammatory diseases. Moreover, VAP-1 may be an alternative therapeutic target in tumors. The in vivo imaging of inflammation using VAP-1 as a target molecule is a novel approach with a potential for early detection and characterization of inflammatory diseases and has obvious clinical significance. Based on the properties and results obtained so far from preclinical and clinical studies, VAP-1 may provide a novel research direction or a potent therapeutic strategy for ophthalmological diseases, including inflammatory lymph- and angiogenic diseases, including cancer.

## Figures and Tables

**Table 1:** The function of vascular adhesion protein-1 in ocular diseases.

Eye diseases	Possible role of VAP-1	Reference
Uveitis	VAP-1 is involved in leukocyte infiltration into ocular tissues.	[11]

NVC	During acute inflammation, VAP-1 regulates both firm adhesion and transmigration; VAP-1 contributes to the recruitment of macrophages to CNV lesions and has a novel link with angiogenesis.	[12, 50]

DR	In chronic low-grade inflammation, VAP-1 may only regulate transmigration; sVAP-1 is increased and correlated with oxidative stress in the vitreous fluid.	[10, 64]

Tumor	VAP-1 is correlated with the angiogenesis and tumor growth.	[71, 72]

VAP-1: vascular adhesion protein-1.
